# The Pre-Treatment Platelet-to-Lymphocyte Ratio as a Prognostic Factor for Loco-Regional Control in Locally Advanced Rectal Cancer

**DOI:** 10.3390/diagnostics13040679

**Published:** 2023-02-11

**Authors:** Richard Partl, Katarzyna Paal, Bettina Stranz, Eva Hassler, Marton Magyar, Thomas Baptist Brunner, Tanja Langsenlehner

**Affiliations:** 1 Department of Therapeutic Radiology and Oncology, Comprehensive Cancer Center, Medical University of Graz, 8036 Graz, Austria; 2 Division of Neuroradiology, Vascular and Interventional Radiology, Comprehensive Cancer Center Graz (CCC), Medical University of Graz, 8036 Graz, Austria

**Keywords:** platelet-to-lymphocyte ratio (PLR), inflammation, prognosis, locally advanced rectal cancer (LARC)

## Abstract

Chronic inflammatory reactions have been proven to represent relevant mechanisms for the development and progression of cancer in numerous tumor entities. There is evidence that the platelet-to-lymphocyte ratio (PLR) is associated with the prognostic outcome. In rectal cancer, the prognostic role of this parameter has not yet been conclusively clarified. The aim of this study was to further clarify the prognostic significance of the pre-treatment PLR in patients with locally advanced rectal cancer (LARC). In the present study, 603 patients with LARC, who were treated with neoadjuvant chemoradiotherapy (nCRT) and subsequent surgical resection between 2004 and 2019, were retrospectively evaluated. The influence of clinico-pathological and laboratory factors on locoregional control (LC), metastasis-free survival (MFS) and overall survival (OS) was investigated. In univariate analyses, high PLR was significantly associated with worse LC (*p* = 0.017) and OS (*p* = 0.008). In multivariate analyses, the PLR remained an independent parameter for the LC (HR = 1.005, 95% CI: 1.000–1.009, *p* = 0.050). Pre-treatment lactate dehydrogenase (LDH) (HR: 1.005 95% CI:1.002–1.008; *p* = 0.001) and carcinoembryonic antigen (CEA) (HR: 1.006, 95% CI:1.003–1.009; *p* < 0.001) were independent predictors for MFS; additionally, age (HR: 1.052, 95% CI:1.023–1.081; *p* < 0.001), LDH (HR: 1.003, 95% CI:1.000–1.007; *p* = 0.029) and CEA (HR: 1.006, 95% CI:1.003–1.009; *p* < 0.001) independently predicted OS. Pre-treatment PLR before nCRT is an independent prognostic factor for LC in LARC, which could be used to further individualize tumor treatment.

## 1. Introduction

A multimodal treatment combining neoadjuvant chemoradiotherapy (nCRT) and surgery is a cornerstone in the treatment of locally advanced rectal cancer (LARC) [1]. Prognosis has been improved due to earlier diagnosis and better treatment modalities [2]. The prediction of treatment outcome is, however, a complex issue involving TNM stage, tumor grade, patient age, and laboratory parameters [3]. Thus, the identification of reliable prognostic factors for treatment outcome is important to improve risk-adapted treatment strategies and subsequent surveillance. Chronic inflammatory response has been identified as a critical component of tumor development, progression, and prognosis in different cancer sites [4,5,6,7]. There is increasing evidence demonstrating an association of systemic inflammation and resistance to radiotherapy and chemotherapy. Several studies have confirmed that C-reactive protein (CRP) and neutrophil-to-lymphocyte ratio (NLR) are predictive markers for treatment response and oncological outcomes in rectal cancer patients [8,9,10,11,12].

In recent years, platelets were identified to play an important role in the development, progression, and outcome of various malignancies due to a complex interplay between platelet-induced tumor growth and tumor cell-induced platelet activation [13,14,15,16,17]. However, the mechanism of the association between thrombocytosis and malignancies is not yet clarified in detail. There are several possible explanations for the association between elevated platelet counts and poor prognosis of malignancies. First, platelets might protect circulating tumor cells from cytolysis, thereby promoting metastasis through the vasculature to distant sites, by surface shielding them from immunological detection, and this seems to be the main mechanism of platelet protection [18]. Second, platelets have shown to promote cancer angiogenesis by releasing angiogenesis regulatory proteins such as vascular endothelial growth factor (VEGF) [17]. In colorectal cancer patients, the VEGF levels are elevated in platelets, and elevated levels correlate with advanced cancer state [19]. Moreover, platelets stimulate angiogenic vessel growth and prevent hemorrhage from the angiogenic vessels, which is promoted by the adhesion function of platelets, as mediated by glycoprotein (GP) Ibα, and these processes could stimulate and potentiate tumor cells to form distant metastases [20]. Additionally, platelets may assist tumor cells in invading to adjacent tissues.

An association between elevated platelet-to-lymphocyte ratio (PLR) and poor prognosis in colorectal cancer has also been published in some studies [21]. However, the results were inconsistent and its impact on the prognostic outcome is not conclusively clarified yet. In meta-analyses using a random effect model to estimate the pooled hazard ratio, PLR had a statistically significant association with the overall survival, but not with disease-free survival [22,23]. In addition, data regarding the prognostic significance of the PLR in LARC are sparse.

The purpose of the current study was to further clarify the prognostic significance of PLR in locally advanced rectal cancer prior to nCRT for loco-regional control (LC), metastases-free survival (MFS), and overall survival (OS) in a large dataset of uniformly treated patients.

## 2. Materials and Methods

### 2.1. Patients and Study Design

In this retrospective single-center study, patients with histologically confirmed LARC (stage II–III) referred for nCRT and subsequent surgical resection in the period between 2004–2019 were selected. The exclusion criteria were (1) missing PLR value, (2) induction chemotherapy prior to nCRT, (3) premature termination of radiotherapy, and (4) neo-adjuvant radiation without concurrent chemotherapy.

### 2.2. Treatment Regime and Follow-Up

Digital rectal examination, pre-treatment colonoscopy, rigid proctoscopy, endorectal ultrasound, and pelvic computed tomography (CT) or magnetic resonance imaging (MRI) was performed to determine clinical tumor stage (cT) and clinical lymph node involvement. Chest and abdomen CT were performed to rule out distant metastases. The distance to the anal verge was measured by means of either colonoscopy or rigid proctoscopy and additional digital rectal examination.

External-beam radiotherapy to the pelvis was applied in prone position with a full bladder and an empty rectum. All patients received radiotherapy in a 3D-conformal 3-4-field technique or intensity-modulated radiotherapy (IMRT), including volumetric modulated arc therapy (VMAT) with photon energies of 6 or 18 MEV up to a total dose of 45–46 Gy in 23–25 fractions of 1.8 or 2 Gy (5 days/week). After a median interval of 6.6 weeks, either a total mesorectal excision (TME) or an abdominoperineal rectum resection (APR) was performed. All patients received concurrent fluoropyrimidine-based chemotherapy that was performed as a continuous intravenous infusion with 5-fluoruracil (1000 mg/m^2^) during the first and last week of radiotherapy or as oral administration of capecitabine (1650 mg/m^2^ daily) on each day of radiation treatment.

The following clinical, pathological, and laboratory parameters, which were documented prior to nRCT, were extracted from medical charts: sex, age at initiation of nRCT, smoking status, tumor site, histopathological tumor grading, clinical tumor stage, clinical lymph node involvement, chemotherapy, serum lactate dehydrogenase (LDH), carcinoembryonic antigen (CEA), carbohydrate antigen 19.9 (CA 19.9), NLR (calculated as the absolute neutrophil count divided by the absolute lymphocyte count) and PLR (calculated as the absolute platelet count divided by the absolute lymphocyte count).

Clinical follow-up examination was conducted in regular intervals by the referring surgeon in accordance with institutional recommendations and by the Department of Therapeutic Radiology and Oncology. Clinical examination, proctoscopy/colonoscopy and abdominal ultrasound were performed twice a year (years 1–2) and once a year (years 3–5). Additional imaging was performed if indicated.

### 2.3. Study Endpoints and Statistical Analysis

The primary endpoint, loco-regional control (LC), was calculated from the start of treatment to the date of local tumor recurrence and/or regional lymph node metastases. Secondary endpoints included metastases-free survival (MFS) defined as the time from the start of treatment to the development of distant metastases, and overall survival (OS) defined as the time from the start of treatment to the date of death of any cause. Continuous data are presented as mean values, and standard deviation or median values and range; categorical data are provided as absolute numbers and relative frequencies.

Univariate and multivariate Cox proportional hazards regression analyses were performed to determine the influence of PLR level and other clinico-pathological and laboratory on LC, MFS and OS. Hazard ratios (HRs) estimated from the Cox proportion analysis are reported as relative risks with corresponding 95% confidence intervals (CIs). Multivariate Cox proportion analyses included parameters with a *p*-value < 0.20 in univariate analyses. Kaplan–Meier methods were used to analyze time-to-event data. A non-parametric stratified log-rank test was used to detect the difference in survival between treatment groups. The association between the PLR and other clinic-pathological features was analyzed by non-parametric tests (Mann–Whitney U Test, Kruskal–Wallis Test, and Spearman correlation).

Receiver operating characteristic (ROC) curve analyses were performed to estimate the optimal PLR cut-point values. The optimal cut-point was determined as the point on the ROC curve that maximizes the Youden Index.

All statistical analyses were performed using the Statistical Package for Social Sciences version 26.0 (SPSS Inc., Chicago, IL, USA). A two-sided *p* < 0.05 was considered statistically significant.

The study complied with the Declaration of Helsinki and was performed in accordance with national law. The study protocol has been approved by the local Ethical Committee. As this was a retrospective non-interventional study, the institutional review board waived the need for written informed consent from the participants.

## 3. Results

Between 2004 and 2019 a total of 603 consecutive patients met the inclusion criteria for further analysis in this study. Patient characteristics are presented in Table 1. Median age at start of nRCT was 66.1 years (mean 65.4 ± 10.6).

The PLR level significantly correlated with clinical tumor stage (cT2-3 vs. 4, *p* < 0.001), clinical nodal involvement (N− vs. N+, *p* = 0.008), clinical stage (II vs. III/IV, *p* = 0.016) and NLR (*p* < 0.001). No significant correlations were observed between the PLR and the remaining clinico-pathological parameters (all *p* > 0.05). A TME was performed in 438 patients (72.6%), and the remaining 165 patients (27.4%) underwent an APR. A complete histopathological response (ypT0 ypN0) was found in 85 patients (14.1%). Residual tumor after nRCT was histopathologically staged as ypT1, ypT2, ypT3, and ypT4 in 7.3%, 27.2%, 49.3%, and 4.3%, respectively. Pathologic nodal stage was diagnosed as ypN0 in 74.7% and ypN+ in 25.3% of the cases.

The median follow-up time was 46.6 months (mean 50.9 ± 32.8 months). During this period, 38 patients (6.3%) developed loco-regional recurrence, 114 patients (18.9%) had distant metastases and 89 patients (14.8%) had died. The 3- and 5- year estimates for LC were 91.9% and 85.4%, the 3- and 5-year estimates for MFS were 76.1% and 60.9%, and the 3- and 5-year OS probabilities were 88.1% and 75.2%, respectively.

In univariate analysis, the pre-treatment PLR level was significantly associated with LC (HR 1.003, 95% CI 1.001–1.006; *p* = 0.017) and OS (HR 1.002 95% CI 1.001–1.004; *p* = 0.008). Univariate analysis also identified the smoking status as a significant prognostic factor for LC (HR 0.449, 95%CI 0.211–0.953, *p* = 0.037). Furthermore, LDH (HR 1.003, 95%CI 1.001–1.006, *p* = 0.007) and CEA (HR 1.007, 95%CI 0.004–0.009, *p* < 0.001) showed a significant relationship with MFS. Additionally, patient age (HR 1.041, 95%CI 1.019–1.064, *p* = 0.001), CEA (HR 1.005, 95%CI 1.003–1.008, *p* < 0.001, and NLR (HR 1.104, 95%CI 1.027–1.186, *p* = 0.007) were significantly associated with OS in univariate analysis. None of the remaining pre-treatment patient and treatment characteristics were significantly associated with LC, MFS and OS in univariate analysis (Table 2).

In multivariate analysis that included parameters with a *p*-value < 0.20 in univariate analysis, the pre-treatment PLR (HR 1.005, 95%CI 1.000–1.009; *p* = 0.050) remained a significant prognostic factor for LC. In addition, multivariate analyses revealed a significant association of LDH (HR 1.005, 95% CI 1.002–1.008; *p* < 0.001), and CEA (HR 1.006 95% CI 1.003–1.009; *p* < 0.001) with MFS. Furthermore, age (HR 1.052, 95% CI 1.023–1.081; *p* < 0.001), LDH (HR 0.003, 95% CI 1.000–1.007; *p* = 0.029), and CEA (HR 1.006, 95% CI 1.003–1.009; *p* > 0.001) were independently associated with OS (Table 3).

**Figure 1:**
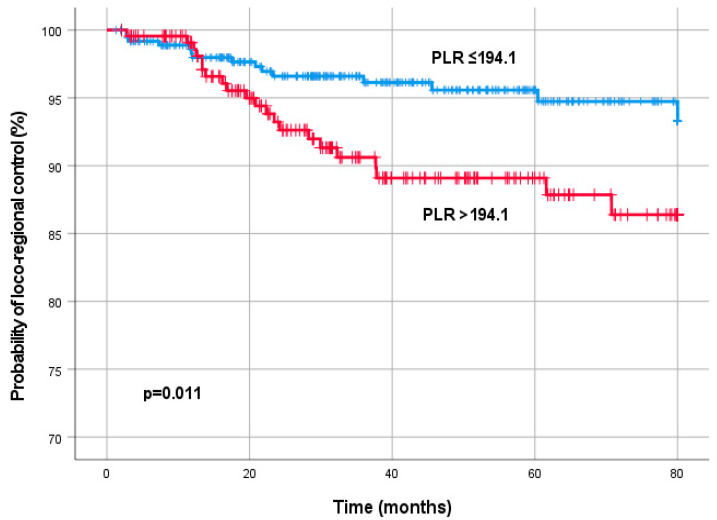
Kaplan–Meier curves for loco-regional control by the pre-treatment platelet-to-lymphocyte ratio (PLR).

Using ROC curve analysis, a pre-treatment PLR cut-off value of 194.1 was determined to be optimal to discriminate LC. Overall, there were 385 patients (63.8%) with PLR ≤194.1, and 218 patients (36.2%) with PLR >194.1. Kaplan–Meier analysis demonstrated a significantly improved LC (*p* = 0.011) for patients with a PLR level ≤194.1 (Figure 1).

**Figure 2:**
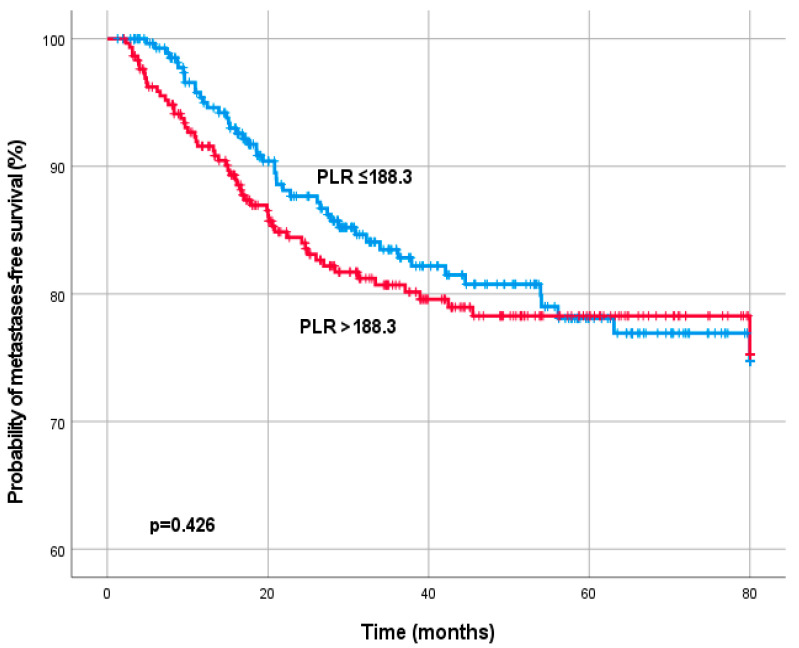
Kaplan–Meier curves for metastases-free survival by the pre-treatment platelet-to-lymphocyte ratio (PLR).

**Figure 3:**
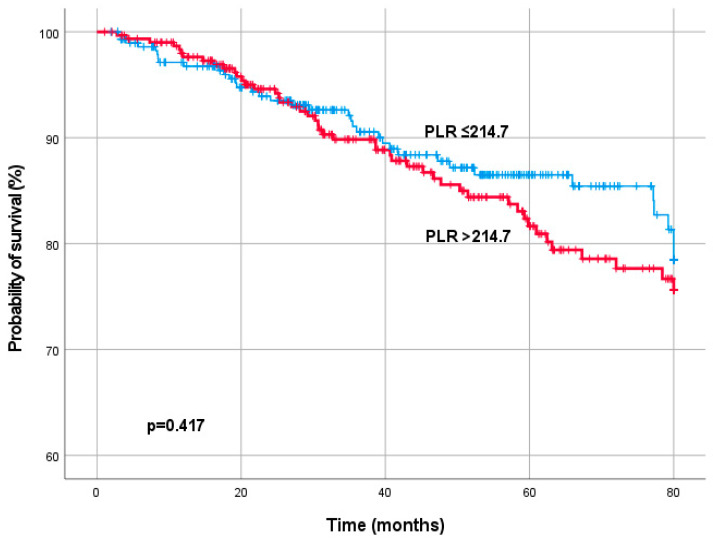
Kaplan–Meier curves for overall survival by the pre-treatment platelet-to-lymphocyte ratio (PLR).

For MFS, ROC curve analysis determined a PLR cut-off value of 188.3. 363 patients (60.2%) had a PLR ≤ 188.3 and 240 patients (39.8%) a PLR > 188.3. For Os, ROC curve analysis determined a PLR cut-off value of 214.7. 417 patients (72.5%) had a PLR ≤ 214.7 and 166 patients (27.5%) a PLR > 214.7. In Log-rank test a significant association between PLR, MFS (*p* = 0.426) and OS (0.417) was not detected (Figure 2 and Figure 3).

## 4. Discussion

Chronic inflammation is a hallmark of cancer, and inflammatory biomarkers are increasingly recognized as prognostic factors for poor clinical outcome [11]. Identification of those prognostic inflammatory biomarkers is a major goal in oncologic studies for better risk stratification and prediction of response to treatment. Recently, particular interest has been focused on the prognostic role of increased platelets for patients with malignancies. The PLR is a cheap and easily measurable ratio of two full blood count components that helps to provide an insight into the inflammatory status and has recently become a potential prognostic factor. An increasing number of studies provide evidence that the PLR is associated with the prognostic outcome in different cancer types [16,21,24,25,26,27,28]. Skandera et al. identified the elevated preoperative PLR to predict the time to recurrence in colon cancer patients [29]. However, data on the prognostic role of the PLR in rectal cancer are limited.

In the present study, we analyzed the prognostic significance of pre-treatment PLR level in 603 patients with LARC and detected a significant association between a high PLR and poor LC, whereas a significant effect on MFS and OS was not found. In addition, higher PLR level significantly correlated with clinical tumor stage 4, clinical nodal involvement, clinical stage III/IV and NLR.

Only a few researchers have previously analyzed the value of pretreatment PLR to predict the outcome in neoadjuvant-treated colorectal cancer. In 145 colorectal cancer patients treated with neoadjuvant chemotherapy a high PLR was an independent parameter for worse OS and short disease-free survival (DFS) [21]. Moreover, in metastatic colorectal cancer PLR >195.5 was an independent prognostic factor for progression-free survival and OS [30]. Furthermore, a retrospective study by Mori et al. enrolled 157 patients with stage I–III colorectal cancer undergoing curative surgery [31]. Kaplan–Meier analysis showed that patients with high PLR was associated with poorer DFS than patients with lower levels. However, this finding could not be validated in Cox’s proportional hazards regression tests. Toiyama et al. retrospectively analyzed the value of the PLR in a cohort of study 89 rectal cancer patients and demonstrated lower OS and disease-free survival (DFS) rates associated with high PLR values, but the differences were not significant [13].

Our findings underline previous studies of the prognostic effect of the pretreatment PLR. This study is the first one in patients treated with preoperative chemoradiation followed by surgery for LARC, suggesting that in a sufficiently larger cohort PLR is correlated with the local recurrence rate. It remains unclear why the impact on metastasis-free survival and overall survival could be identified in previous colorectal studies, but not in our large rectal cancer cohort treated with nCRT. A recent study with a comparable patient cohort of 100 patient supports our findings, that PLR in LARC is not correlated with OS [32]. Interestingly, in large meta-analysis in advanced and metastatic non-small-cell lung cancer elevated PLR is also not associated with OS and progression-free survival, suggesting that PLR may be not a reasonable biomarker in this subset [33]. More studies are preferred to further validate the effect of PLR for metastasis-free survival and overall survival in patients with advanced colorectal cancer.

In most studies a significant heterogeneity for the optimal cut-off point can be observed, ranging between 150 and 300 [23]. More recent studies indicate a lower value based on ROC analysis, ranging between 25.4 and 154.3 [21,34]. In our study, the optimal cut-off value was 194.1 for the primary endpoint LC.

Major strengths of our study are the well-defined study cohort and the fact that the radiotherapy was planned and administered in a consistent manner throughout a period of 15 years. To the best of our knowledge, the present study is currently the largest one investigating the prognostic value of the PLR in patients with LARC. Nevertheless, some limitations for this analysis must be taken into account. First, because of its retrospective design collected from a single institution, we are unable to exclude the possibility of an unequal distribution of unidentified clinicopathologic parameters in our cohort that may have biased the observed results. Validation of our data in additional prospective studies with enough statistical power is imperative before firm conclusions can be drawn. Second, the value of the PLR is also influenced by pathogenic and non-pathogenic related inflammatory reactions. In acute and chronic liver disease associated with hepatitis B virus, platelets are known to be involved by upregulating migration of virus-specific CD8+ T cells and non-specific inflammatory cells into the liver [35]. In a non-pathogenic context, an interaction between platelets, endothelial cells and white blood cells in atherosclerosis has been demonstrated [36]. Furthermore, several agents such as nonsteroidal anti-inflammatory drugs, antibiotics and cardiovascular and lipid-lowering drugs can impair platelet function. These interactions should also be taken into consideration. Third, information about second malignancies was not completely available. Fourth, the calculated cut-off values in our data set were different from those in previous studies.

Nevertheless, the present study demonstrated that the PLR could act as an inexpensive objective biomarker in the prediction of LC in locally advanced rectal cancer. If validated in further studies, the PLR might help to stratify patient in daily oncologic clinical practice and clinical trials.

## Figures and Tables

**Table 1:** Patient characteristics.

Parameter	Value
Number of patients	603
Sex	
Male Female	406 (67.3%) 197 (32.7%)
Age; median (mean ± SD)	66.6 (65.6 ± 10.5)
Smoking status	
Current Former* or never Missing	84 (13.9%) 370 (61.4%) 149 (24.7%)
Tumor site	
<6 cm ab ano 6–12 cm ab ano 12–16 cm ab ano	344 (57.0%) 241 (40.0%) 18 (3.0%)
Tumor grade	
G 1 G 2 G 3 Missing	41 (6.8%) 515 (85.4%) 42 (7.0%) 5 (0.8%)
Clinical tumor stage	
T2 T3 T4	16 (2.7%) 522 (86.6%) 65 (10.7%)
Clinical nodal involvement	
Yes No	370 (61.4%) 233 (38.6%)
Clinical stage	
Stage II Stage III/IV	228 (37.8%) 375 (62.2%)
Chemotherapy	
5-Fluoruracil Cabecitabine Missing	451 (74.8%) 151 (25.0%) 1 (0.2%)
LDH, median (mean ± SD)	178.5 (193.4 ± 67.0)
CEA, median (mean ± SD)	3.5 (9.9 ± 39.2)
CA 19-9, median (mean ± SD)	8.1 (27.0 ± 84.5)
NLR, median (mean ± SD)	3.1 (3.6 ± 2.1)
PLR, median (mean ± SD)	173.9 (193.9 ± 95.9)

* Former smoking was defined as tobacco use before or until the start of treatment. Abbreviations: LDH, lactate dehydrogenase; CEA, carcinoembryonic antigen; CA 19-9, carbohydrate antigen; NLR, neutrophil-to-lymphocyte ratio; PLR, platelet-to-lymphocyte ratio; SD, standard deviation.

**Table 2:** Univariate analysis of clinico-pathological and laboratory factors for the prediction of loco-regional control, metastases-free survival and overall survival.

	Loco-Regional Control		Metastases-Free Survival		Overall Survival	
Criterion	HR (95% CI)	*p*-Value	HR (95% CI)	*p*-Value	HR (95% CI)	*p*-Value
Sex						
Male Female	1 0.656 (0.309–1.390)	0.271	1 1.191 (0.799–1.775)	0.392	1 1.782 (0.492–1.241)	0.296
Age (continuous)	0.987 (0.957–1.017)	0.390	1 1.008 ((0.989–1.028)	0.389	1.041 (1.019–1.064)	< 0.001
Smoking status						
Current Former/Never	1 0.449 (0.211–0.953)	0.037	1 0.791 (0.455–1.373)	0.404	1 1.216 (0.621–2.238)	0.568
Tumor site (ab ano)						
<6 cm 6–12 cm 12–16 cm	1 0.707 (0.360–1.389) 0 (0–1.358)	0.314 0.974	1 1.191 (0.807–1.757) 0.593 (0.145–2.436)	0.379 0.469	1 1.127 (0.740–1.718) 0.712 (0.173–2.933)	0.577 0.638
Tumor grade						
G 1 G 2 G 3	1 2.664 (0.364–19.488) 2.015 (0.183–22.226)	0.334 0.567	1 1.470 (0.597–3.618) 1.794 (0.601–5.355)	0.402 0.295	1 1.035 (0.451–2.378) 1.209 (0.406–3.599)	0.935 0.733
Tumor stage						
T 1-3 T 4	1 1.585 (0.617–4.072)	0.338	1 1.468 (0.820–2.625)	0.196	1 1.583 (0.861–2.910)	0.139
Nodal involvement						
No Yes	1 1.634 (0.807–3.309)	0.172	1 1.201 (0.805–1.790)	0.369	1 0.871 (0.573–1.323)	0.517
Clinical stage						
II III/IV	1 1.414 (0.710–2.816)	0.324	1 1.305 (0.871–1.955)	0.197	1 0.989 (0.649–1.508)	0.959
Chemotherapy						
5-Fluoruracil Cabecitabine	1 0.970 (0.458–2.057)	0.938	1 0.854 (0.538–1.358)	0.505	1 1.111 (0.696–1.774)	0.660
LDH (continuous)	0.996 (0.990–1.003)	0.263	1.003 (1.001–1.006)	0.007	1.002 (1.000–1.005)	0.108
CEA (continuous)	1.003 (0.997–1.009)	0.407	1.007 (1.004–1.009)	< 0.001	1.005 (1.003–1.008)	< 0.001
CA 19-9 (continuous)	1.002 (0.999–1.004)	0.147	1.001 (0.999–1.003)	0.375	1.000 (0.998–1.003)	0.819
NLR (continuous)	1.083 (0.966–1.214)	0.173	1.034 (0.951–1.124)	0.436	1.104 (1.027–1.186)	0.007
PLR (continuous)	1.003 (1.001–1.006)	0.017	1.001 (0.999–1.003)	0.446	1.002 (1.001–1.004)	0.008

**Table 3:** Multivariate analysis of clinico-pathological and laboratory factors for the prediction of loco-regional control, metastases-free survival and overall survival.

	Loco-Regional Control		Metastases-Free Survival		Overall Survival	
Criterion	HR (95% CI)	*p*-Value	HR (95% CI)	*p*-Value	HR (95% CI)	*p*-Value
Age (continuous)					1.052 (1.023–1.081)	<0.001
Smoking status						
Current Former/Never	1 0.720 (0.250–2.077)	0.544				
Tumor stage						
T 1-3 T 4			1 1.574 (0.786–3.150)	0.200	1 1.581 (0.703–3.557)	0.268
Nodal involvement						
No Yes	1 1.275 (0.505–3.219)	0.607				
Clinical stage						
II III/IV			1 1.353 (0.814–2.249)	0.243		
LDH (continuous)			1.005 (1.002–1.008)	<0.001	1.003 (1.000–1.007)	0.029
CEA (continuous)			1.006 (1.003–1.009)	<0.001	1.006 (1.003–1.009)	<0.001
CA 19-9 (continuous)	1.002 (0.999–1.004)	0.196				
NLR (continuous)	0.947 (0.741–1.210)	0.662			0.938 (0.798–1.102)	0.435
PLR (continuous)	1.005 (1.000–1.009)	0.050			1.002 (0.999–1.006)	0.205

## Data Availability

The data presented in this study are available on request from the corresponding author. The data are not publicly available due to ethical restrictions.

## References

[1] Benson A.B., Venook A.P., Al-Hawary M.M., Arain M.A., Chen Y.J., Ciombor K.K., Cohen S., Cooper H.S., Deming D., Garrido-Laguna I. (2020). NCCN Guidelines Insights: Rectal Cancer, Version 6.2020: Featured Updates to the NCCN Guidelines. J. Natl. Compr. Cancer Netw..

[2] Siegel R., Miller K., Jemal A. (2020). Cancer statistics, 2020. CA Cancer J. Clin..

[3] Argilés G., Tabernero J., Labianca R., Hochhauser D., Salazar R., Iveson T., Laurent-Puig P., Quirke P., Yoshino T., Taieb J. (2020). Localised colon cancer: ESMO Clinical Practice Guidelines for diagnosis, treatment and follow-up†. Ann. Oncol..

[4] Karki R., Man S., Kanneganti T.-D. (2017). Inflammasomes and Cancer. Cancer Immunol. Res..

[5] Sciarra A., Gentilucci A., Salciccia S., Pierella F., Del Bianco F., Gentile V., Silvestri I., Cattarino S. (2016). Prognostic value of inflammation in prostate cancer progression and response to therapeutic: A critical review. J. Inflamm..

[6] Grivennikov S.I., Greten F., Karin M. (2010). Immunity, inflammation, and cancer. Cell.

[7] Makita K., Hamamoto Y., Takata N., Ishikawa H., Tsuruoka S., Uwatsu K., Hato N., Kido T. (2021). Prognostic significance of inflammatory response markers for locally advanced squamous cell carcinoma of the external auditory canal and middle ear. J. Radiat. Res..

[8] Jones H.G., Qasem E., Dilaver N., Egan R., Bodger O., Kokelaar R., Evans M.D., Davies M., Beynon J., Harris D. (2018). Inflammatory cell ratios predict major septic complications following rectal cancer surgery. Int. J. Color. Dis..

[9] Shen J., Zhu Y., Wu W., Zhang L., Ju H., Fan Y., Zhu Y., Luo J., Liu P., Zhou N. (2017). Prognostic role of neutrophil-to-lymphocyte ratio in locally advanced rectal cancer treated with neoadjuvant chemoradiotherapy. Med. Sci. Monit. Int. Med. J. Exp. Clin. Res..

[10] Zhang Y., Liu X., Xu M., Chen K., Li S., Guan G. (2020). Prognostic value of pretreatment systemic inflammatory markers in patients with locally advanced rectal cancer following neoadjuvant chemoradiotherapy. Sci. Rep..

[11] Naszai M., Kurjan A., Maughan T. (2021). The prognostic utility of pre-treatment neutrophil-to-lymphocyte-ratio (NLR) in colorectal cancer: A systematic review and meta-analysis. Cancer Med..

[12] Partl R., Lukasiak K., Thurner E.-M., Renner W., Stranzl-Lawatsch H., Langsenlehner T. (2020). The Elevated Pre-Treatment C-Reactive Protein Predicts Poor Prognosis in Patients with Locally Advanced Rectal Cancer Treated with Neo-Adjuvant Radiochemotherapy. Diagnostics.

[13] Toiyama Y., Inoue Y., Kawamura M., Kawamoto A., Okugawa Y., Hiro J., Saigusa S., Tanaka K., Mohri Y., Kusunoki M. (2015). Elevated platelet count as predictor of recurrence in rectal cancer patients undergoing preoperative chemoradiotherapy followed by surgery. Int. Surg..

[14] Kawai K., Kitayama J., Tsuno N.H., Sunami E., Watanabe T. (2013). Thrombocytosis before pre-operative chemoradiotherapy predicts poor response and shorter local recurrence-free survival in rectal cancer. Int. J. Color. Dis..

[15] Lin R.J., Afshar-Kharghan V., Schafer A. (2014). Paraneoplastic thrombocytosis: The secrets of tumor self-promotion. Blood.

[16] Wang C., Tong J., Tang M., Lu Y., Liang G., Zhang Z., Chen T. (2021). Pretreatment Neutrophil-to-Lymphocyte Ratio and Platelet-to-Lymphocyte Ratio as Prognostic Factors and Reference Markers of Treatment Options for Locally Advanced Squamous Cell Carcinoma Located in the Middle and Upper Esophagus. Cancer Manag. Res.

[17] Tesfamariam B. (2016). Involvement of platelets in tumor cell metastasis. Pharmacol. Ther..

[18] Nieswandt B., Hafner M., Echtenacher B., Männel D.N. (1999). Lysis of tumor cells by natural killer cells in mice is impeded by platelets. Cancer Res..

[19] Peterson J.E., Zurakowski D., Italiano J.E., Michel L.V., Connors S., Oenick M., D’Amato R.J., Klement G.L., Folkman J. (2012). VEGF, PF4 and PDGF are elevated in platelets of colorectal cancer patients. Angiogenesis.

[20] Kisucka J., Butterfield C.E., Duda D.G., Eichenberger S.C., Saffaripour S., Ware J., Ruggeri Z.M., Jain R.K., Folkman J., Wagner D.D. (2006). Platelets and platelet adhesion support angiogenesis while preventing excessive hemorrhage. Proc. Natl. Acad. Sci. USA.

[21] Jia W., Yuan L., Ni H., Xu B., Zhao P. (2021). Prognostic Value of Platelet-to-Lymphocyte Ratio, Neutrophil-to-Lymphocyte Ratio, and Lymphocyte-to-White Blood Cell Ratio in Colorectal Cancer Patients Who Received Neoadjuvant Chemotherapy. Technol. Cancer Res. Treat..

[22] Zhang J., Zhang H.-Y., Li J., Shao X.-Y., Zhang C.-X. (2017). The elevated NLR, PLR and PLT may predict the prognosis of patients with colorectal cancer: A systematic review and meta-analysis. Oncotarget.

[23] Tan D., Fu Y., Su Q., Wang H. (2016). Prognostic role of platelet-lymphocyte ratio in colorectal cancer: A systematic review and meta-analysis. Medicine.

[24] Zhou X., Du Y., Huang Z., Xu J., Qiu T., Wang J., Wang T., Zhu W., Liu P. (2014). Prognostic value of PLR in various cancers: A meta-analysis. PLoS ONE.

[25] Ding N., Pang Z., Shen H., Ni Y., Du J., Liu Q. (2016). The Prognostic Value of PLR in Lung Cancer, a Meta-analysis Based on Results from a Large Consecutive Cohort. Sci. Rep..

[26] Langsenlehner T., Pichler M., Thurner E.-M., Krenn-Pilko S., Stojakovic T., Gerger A., Langsenlehner U. (2015). Evaluation of the platelet-to-lymphocyte ratio as a prognostic indicator in a European cohort of patients with prostate cancer treated with radiotherapy. Urol. Oncol. Semin. Orig. Investig..

[27] Zhou K., Cao J., Lin H., Liang L., Shen Z., Wang L., Peng Z., Mei J. (2022). Prognostic role of the platelet to lymphocyte ratio (PLR) in the clinical outcomes of patients with advanced lung cancer receiving immunotherapy: A systematic review and meta-analysis. Front. Oncol..

[28] Chen H., Wu X., Wen Z., Zhu Y., Liao L., Yang J. (2022). The Clinicopathological and Prognostic Value of NLR, PLR and MLR in Non-Muscular Invasive Bladder Cancer. Arch. Esp. Urol..

[29] Szkandera J., Pichler M., Absenger G., Stotz M., Arminger F., Weissmueller M., Schaberl-Moser R., Samonigg H., Kornprat P., Stojakovic T. (2014). The elevated preoperative platelet to lymphocyte ratio predicts decreased time to recurrence in colon cancer patients. Am. J. Surg..

[30] Acikgoz O., Cakan B., Demir T., Bilici A., Oven B.B., Hamdard J., Olmuscelik O., Olmez O.F., Seker M., Yildiz O. (2021). Platelet to lymphocyte ratio is associated with tumor localization and outcomes in metastatic colorectal cancer. Medicine.

[31] Mori K., Toiyama Y., Saigusa S., Fujikawa H., Hiro J., Kobayashi M., Ohi M., Araki T., Inoue Y., Tanaka K. (2015). Systemic Analysis of Predictive Biomarkers for Recurrence in Colorectal Cancer Patients Treated with Curative Surgery. Dig. Dis. Sci..

[32] Duque-Santana V., López-Campos F., Martin-Martin M., Valero M., Zafra-Martín J., Couñago F., Sancho S. (2022). Neutrophil-to-lymphocyte ratio and platelet-to-lymphocyte ratio as prognostic factors in locally advanced rectal cancer. Oncology.

[33] Li B., Zhou P., Liu Y., Wei H., Yang X., Chen T., Xiao J. (2018). Platelet-to-lymphocyte ratio in advanced Cancer: Review and meta-analysis. Clin. Chim. Acta.

[34] Ozawa T., Ishihara S., Nishikawa T., Tanaka T., Tanaka J., Kiyomatsu T., Hata K., Kawai K., Nozawa H., Kazama S. (2015). The preoperative platelet to lymphocyte ratio is a prognostic marker in patients with stage II colorectal cancer. Int. J. Color. Dis..

[35] Aiolfi R., Sitia G. (2015). Chronic hepatitis B: Role of anti-platelet therapy in inflammation control. Cell. Mol. Immunol..

[36] Davi G., Patrono C. (2007). Platelet activation and atherothrombosis. N. Engl. J. Med..

